# CT Head Imaging Within 60 Minutes of Arrival at the ED: A Clinical Audit

**DOI:** 10.7759/cureus.92764

**Published:** 2025-09-20

**Authors:** Osman S Elhassan, Mohammed Qasim Rauf, Omar Mustafa Odeh Odeh, Kirsty Farrell, Bilal Al-Obaidi, Anshul Sobti

**Affiliations:** 1 Trauma and Orthopaedics, and Emergency Medicine, The Hillingdon Hospitals NHS Foundation Trust, London, GBR; 2 Trauma and Orthopaedics, The Hillingdon Hospitals NHS Foundation Trust, London, GBR; 3 Trauma and Orthopaedics, Imperial College Healthcare NHS Trust, London, GBR; 4 Emergency Medicine, The Hillingdon Hospitals NHS Foundation Trust, London, GBR

**Keywords:** audit, ct head, emergency department, head injury, nice guidelines, pro forma, triage

## Abstract

Objective

This audit aimed to assess current compliance with the National Institute for Health and Care Excellence (NICE) NG232 guideline and evaluate the impact of introducing a triage pro forma to nurses to improve the time from patients’ arrival in the Emergency Department (ED) to CT head imaging in eligible patients.

Method

A retrospective clinical audit was conducted over two cycles at the ED of a UK District General Hospital. It included patients at the ED who underwent CT head imaging. Non-trauma-related indications and patients under the age of 16 years were excluded. The first cycle, using Electronic Patient Records (EPR), reviewed 138 patients from 20 September 2023 to 20 October 2023. A pro forma was then introduced to assist triage nurses in identifying eligible patients, ensuring they were promptly assessed by a clinician, and imaging was requested. Daily educational sessions were conducted from 20 October 2023 to 3 November 2023, along with email dissemination and handover reinforcement. The second cycle was conducted from 3 November 2023 to 15 November 2023 and included 87 patients, with their data collected from EPR.

Results

In the first cycle, 4.34% (n=6) of patients received imaging within 60 minutes, which is suboptimal. In the second cycle, the proportion of imaging completed within 60 minutes increased to 6.9% (n=6), and this incremental increase is significant (p <0.05).

Conclusion

The introduction of the triage pro forma resulted in incremental improvements in timely brain imaging, and, therefore, the intervention showed significant positive potential in enhancing adherence to national guidelines and improving patient outcomes. Challenges such as staff compliance, patient transfer time, and workload do influence this, and those are areas that need further improvement. This project has the potential to be implemented across multiple trusts.

## Introduction

Head injuries are time-critical emergencies. In Europe, traumatic brain injury (TBI) is a leading cause of mortality and hospitalisation [1]. The National Institute for Health and Care Excellence (NICE) guidelines (NG232) emphasise the importance of CT head imaging within 60 minutes for patients with suspected TBI [2]. A study by Schellenberg et al. found that patients who received prompt CT head imaging, followed by necessary neurosurgical intervention, experienced more favourable functional outcomes than those whose imaging was delayed. Their findings support the clinical importance of early diagnostic imaging in influencing the trajectory of recovery for patients with TBI [3]. 

A greater focus on enhancing compliance with national guidelines could significantly reduce the substantial financial resources currently allocated to the acute care phases for head injuries and have a significant impact on the outcomes of care for these injuries [4]. Research indicates that providing appropriate and timely medical intervention to patients with suspected TBI can significantly enhance their overall outcomes and prognosis [5]. Prompt diagnosis and early management are particularly critical in preventing secondary brain injury and improving survival rates. There is evidence to suggest that quality improvement approaches have been demonstrated to enhance compliance with NICE recommendations, which results in more appropriate utilisation of CT scans and improvement in time to imaging. Improvements in scanning and reporting efficiency have been linked to these measures [6,7]. 

The CT head imaging is widely regarded as a reliable imaging modality for identifying the majority of the hemorrhagic complications associated with head trauma [8]. At our Emergency Department (ED), anecdotal observations and findings from previous audit cycles consistently highlighted suboptimal adherence to the nationally recommended standard for timely CT head imaging in patients with suspected TBI. The NICE guidelines specifically recommend that CT head imaging be performed within 60 minutes for patients meeting certain clinical criteria [2]. Despite the clarity of this recommendation, various systemic and operational factors contributed to delays in achieving this target within our department. 

Among the identified barriers, a significant contributor was the delay in initial clinical assessment by an ED clinician. In many cases, patients who met the criteria for urgent CT head imaging were not promptly recognised at triage, leading to a delay in clinician review and, consequently, in imaging. Recognising the need for early identification, we sought to address this issue by shifting a portion of the decision-making responsibility to triage-level nurses. The intervention involved empowering triage nurses to proactively identify patients at risk of TBI using a structured triage pro forma developed in accordance with NICE criteria. This form was designed to standardise the assessment process and facilitate early recognition of patients requiring urgent imaging. The primary aim of the audit was to assess current compliance with NICE guidelines and evaluate the effectiveness of the triage pro forma and educational initiative in reducing time to CT head imaging for patients with suspected TBI.

## Materials and methods

A retrospective clinical audit was conducted at the ED of the Hillingdon Hospital in London, a District General Hospital in the UK, to assess compliance with NICE NG232 guidelines on the timing and indications for CT head imaging in patients presenting with head trauma. Patient records were reviewed over a defined audit period to evaluate whether imaging was performed within the recommended timeframes. The audit was registered with the audit department (registration no. 1610) and did not require Institutional Review Board (IRB) approval.

Study design 

The study was conducted over two audit cycles. The first cycle was conducted from September 20, 2023, to October 20, 2023, to evaluate current practices and establish a reference point before introducing the intervention. The second cycle took place between March 11, 2023, and November 15, 2023, after the intervention was implemented. This design allowed us to compare the figures in the pre- and post-intervention phases.

Inclusion and exclusion criteria 

All adult patients presenting to the ED who underwent CT head imaging for trauma-related indications were included in the study. Patients undergoing CT head imaging for non-trauma-related indications and patients under the age of 16 years were excluded from the study.

Data collection and analysis

Data were obtained from a combination of Electronic Patient Records (EPR) and records from the Radiology Department. Information, including the time of arrival at the ED and the time of CT head imaging, was extracted. These data were entered into Excel spreadsheets (Microsoft Corp., Redmond, WA, USA), and the time from arrival to CT imaging was calculated for each patient (see Appendix A).

A total of 138 patients were included in the first cycle, and 87 patients in the second cycle. Between the two cycles, an education phase was carried out from October 20, 2023, to November 3, 2023, to support the implementation of a structured nurse-led triage pro forma. Comparative analysis between the two audit cycles was conducted using the t-test, with p-values calculated to determine statistical significance. 

Intervention 

**Figure 1:**
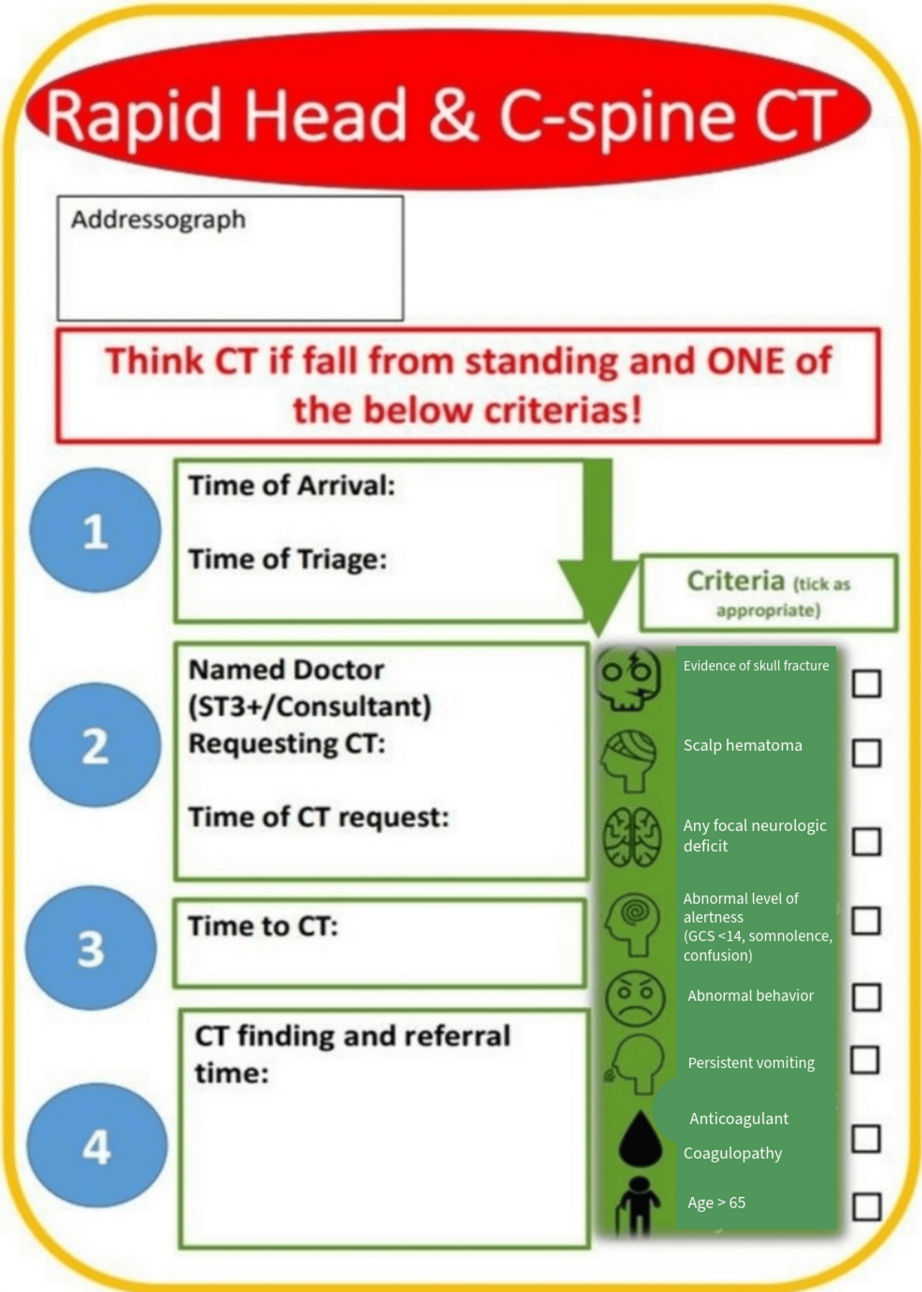
The triage pro forma for the identification and initial management of suspected TBI in accordance with NICE guidelines TBI: Traumatic brain injury, NICE: National Institute for Health and Care Excellence

A structured triage pro forma (Figure 1) was introduced to support the early recognition of patients meeting the criteria for urgent CT head imaging in line with NICE guidelines. This tool served as a prompt for triage nurses to identify eligible patients upon arrival. 

This pro forma was readily available to triage nurses in the triage room. Once a suitable patient was recognised, the triage nurse would fill out the pro forma with what applies to the patient’s case and then immediately notify a clinician, who could then proceed to request a CT head scan without waiting for the standard clinical review pathway. To ensure consistent and accurate implementation, triage nurses received daily face-to-face briefings, with additional reinforcement provided via email updates and reminders during shift handovers. The primary aim of the pro forma was to expedite the clinical assessment process by minimising unnecessary delays, particularly the time taken for patients to be formally reviewed by an ED clinician before imaging could be initiated. 

## Results

Results

The first audit cycle data showed that only six out of 138 patients (4.34%) who had suspected TBI received CT head imaging within the 60-minute window as per NICE NG232 guidelines (Table 1). The low compliance rate indicated a major discrepancy between national standards and actual clinical practice in our department, which required specific interventions to enhance imaging timeliness for this urgent patient population. 

**Table 1:** Results of the first audit cycle The data is categorised into three time intervals and shows the corresponding proportion of patients in each category.

Parameter	Value
Number of scans (n)	138
Within 60 minutes	4.34%
60-120 minutes	19%
Over 120 minutes	76.66%

In the second audit cycle (Table 2), post the implementation of a structured triage pro forma and education-based intervention, six out of 87 patients (6.9%) received their CT head imaging within 60 minutes of arrival. Although the percentage improvement in compliance appeared modest, it is important to consider the context of this change. The absolute number of patients receiving timely imaging remained the same, despite the total number of eligible patients being considerably lower in the second cycle. Therefore, the increase was in fact in the percentage of patients. 

**Table 2:** Results of the second audit cycle The data is categorised into three time intervals and shows the corresponding proportion of patients in each category.

Parameter	Value
Number of scans (n)	87
Within 60 minutes	6.9%
60-120 minutes	16%
Over 120 minutes	77.1%

Furthermore, maintaining the same number of timely CT head imaging despite the smaller cohort may reflect a shift in departmental culture and prioritisation of early imaging. It also implies that the intervention may have contributed to more efficient patient identification and workflow management during periods of increased demand. 

## Discussion

This audit evaluated the time from patients’ arrival in the ED to CT head image acquisition in patients with head injuries, with a specific focus on compliance with the 60-minute target outlined by NICE NG232 [2]. Data from the first cycle showed suboptimal compliance, with only 4.34% of patients scanned within 60 minutes. Following the introduction of a nurse-led triage pro forma, followed by educational interventions, compliance improved to 6.9% in the second cycle. Although this change was modest in percentage terms, it represents a meaningful improvement in absolute patient numbers with potentially improved outcomes. It also shows that low-resource interventions can have a positive impact on diagnostic timeliness in acute care settings. 

While there is evidence to suggest that most head injury patients who present to the ED are not admitted [9], TBI is not a diagnosis to be missed due to the significant risk of mortality and morbidity [10]. We argue that the structured pro forma provides clarity and consistency, reducing variability in decision-making and ensuring that key clinical signs and symptoms are not overlooked. Additionally, it can also be argued that involving nurses in the early stages of triage gives a sense of ownership and accountability, contributing to more streamlined patient flow. When combined with tailored educational sessions, staff are better equipped to recognise indications for imaging and apply guidelines confidently and accurately. Ultimately, these measures support a more coordinated and efficient care pathway, where clinical decisions are aligned with guidelines from the outset.

While some studies have shown nurses need further training and knowledge to triage, we believe that educational sessions delivered specifically on assessment of the signs and symptoms outlined by NICE NG232 can sufficiently enable them to safely assess patients with suspected TBI [11,2]. It has also been demonstrated that the use of standardised assessment pro formas can significantly enhance the documentation process [12,13]. Previously published studies show that improving compliance with NICE guidelines can reduce unnecessary CT requests [14]. Similar studies show that simple interventions can improve compliance with NICE guidelines for ordering CT head scans [15]. This study demonstrated that a well-crafted triage tool that is in line with national guidelines, combined with education, can lead to significant and measurable improvement in compliance with national standards. 

Limitations 

Several limitations must be acknowledged. First, the data collection was retrospective, relying on EPR, which may be subject to documentation errors or time entry inaccuracies. Additionally, this project did not assess clinical outcomes, such as time to definitive intervention or morbidity/mortality, which could strengthen the case for wider adoption. Another limitation of this study is that potential adverse events related to the use of the triage pro forma, such as delays in assessing other injuries or prolonged triage times, were not predefined as measurement items or formally evaluated. Finally, factors such as ED crowding, scanner availability, and variations in staffing were not controlled for, which may have influenced the results. Future cycles could benefit from a longer follow-up period, expanded sample size, and more robust evaluation of outcome measures beyond timing metrics alone. 

## Conclusions

Introducing a simple triage tool combined with structured education improved the timeliness of CT imaging of head injury patients. The modest improvement suggests early benefits, but barriers remain, including transfer delays to radiology, staff compliance and pro forma uptake, and workload pressures during peak times. Future work should focus on sustained education, regular re-auditing, and collaboration with radiology to address operational bottlenecks. This project has the potential to be replicated in other healthcare settings. 

## Figures and Tables

**Figure 2:**
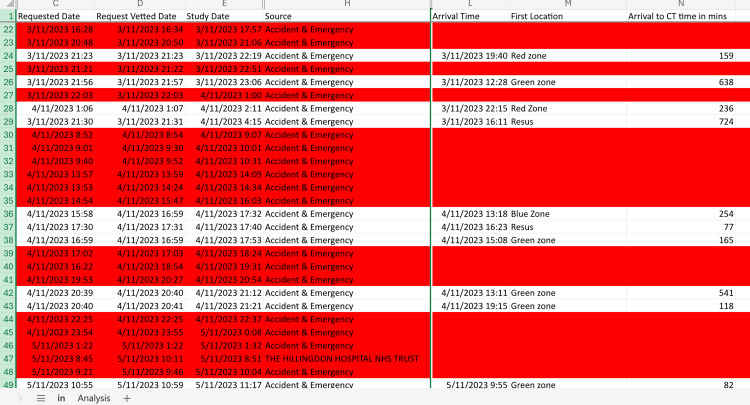
Raw data extracted from the EPR EPR: Electronic Patient Records

## Appendices

Appendix A

Figure 2 features the raw data extracted from the EPR, including the time of arrival at the ED and the time of CT head imaging.
